# Prefrontal Gamma Oscillations Encode Tonic Pain in Humans

**DOI:** 10.1093/cercor/bhv043

**Published:** 2015-03-08

**Authors:** Enrico Schulz, Elisabeth S. May, Martina Postorino, Laura Tiemann, Moritz M. Nickel, Viktor Witkovsky, Paul Schmidt, Joachim Gross, Markus Ploner

**Affiliations:** 1 Department of Neurology, Technische Universität München, 81675 Munich, Germany; 2 TUM - Neuroimaging Center, Technische Universität München, 81675 Munich, Germany; 3 Department of Theoretical Methods, Institute of Measurement Science, Slovak Academy of Sciences, 84219 Bratislava, Slovak Republic; 4 Centre for Cognitive Neuroimaging, Department of Psychology, University of Glasgow, Glasgow G12 8QB, UK

**Keywords:** electroencephalography, gamma oscillations, ongoing pain, pain perception, prefrontal cortex

## Abstract

Under physiological conditions, momentary pain serves vital protective functions. Ongoing pain in chronic pain states, on the other hand, is a pathological condition that causes widespread suffering and whose treatment remains unsatisfactory. The brain mechanisms of ongoing pain are largely unknown. In this study, we applied tonic painful heat stimuli of varying degree to healthy human subjects, obtained continuous pain ratings, and recorded electroencephalograms to relate ongoing pain to brain activity. Our results reveal that the subjective perception of tonic pain is selectively encoded by gamma oscillations in the medial prefrontal cortex. We further observed that the encoding of subjective pain intensity experienced by the participants differs fundamentally from that of objective stimulus intensity and from that of brief pain stimuli. These observations point to a role for gamma oscillations in the medial prefrontal cortex in ongoing, tonic pain and thereby extend current concepts of the brain mechanisms of pain to the clinically relevant state of ongoing pain. Furthermore, our approach might help to identify a brain marker of ongoing pain, which may prove useful for the diagnosis and therapy of chronic pain.

## Introduction

Pain translates objective sensory information into a subjective percept, which signals threat and thereby fulfills vital protective functions. However, pain can also occur as an ongoing percept without obvious sensory information. In such chronic pain states, pain no longer serves a protective function, but represents a pathological condition with devastating effects on quality of life. About a fifth of the adult population suffers from chronic pain, and treatment of these patients is often difficult and unsatisfactory (Breivik et al. 2006).

Our understanding of the brain mechanisms of pain is, however, largely based on studies investigating the processing of brief pain stimuli with a duration of milliseconds to seconds. Functional imaging work has revealed that such stimuli activate an extended network of brain areas including somatosensory, insular, cingulate, and prefrontal cortices (Tracey and Mantyh 2007; Apkarian et al. 2013; Garcia-Larrea and Peyron 2013). Neurophysiological recordings have specified that these brain areas generate different neural responses at frequencies from 3 to 100 Hz, that is, from theta to gamma frequencies, which represent different steps in the translation of objective sensory information into a subjective percept (Garcia-Larrea et al. 2003; Mouraux et al. 2003; Ploner et al. 2006a, 2006b; Gross et al. 2007; Hauck et al. 2007; Schulz et al. 2011; Zhang et al. 2012).

In contrast, the cerebral encoding of ongoing, that is, tonic or chronic pain and whether and how it differs from the encoding of brief pain stimuli is far less well understood. The few existing functional imaging studies indicated that ongoing pain activates similar brain regions as do brief experimental stimuli (Di Piero et al. 1994; Hsieh et al. 1995; Derbyshire and Jones 1998; Schreckenberger et al. 2005; Owen et al. 2010; Wasan et al. 2011). More recent studies have revealed that ongoing pain particularly engages the medial prefrontal cortex (Baliki et al. 2006, 2011; Hashmi et al. 2013), which has been interpreted as a shift away from sensory to emotional processes when pain is ongoing for months and years (Hashmi et al. 2013). Likewise, the neurophysiological encoding of ongoing pain is largely undetermined. Some studies have observed a decrease of neuronal oscillations at alpha frequencies, that is, at around 10 Hz (Chen and Rappelsberger 1994; Ferracuti et al. 1994; Chang et al. 2002; Dowman et al. 2008; Nir et al. 2012; Shao et al. 2012; Peng et al. 2014). A few other investigations have found increases in the amplitude of gamma oscillations (30–100 Hz) (Veerasarn and Stohler 1992; Dowman et al. 2008; Peng et al. 2014). However, these neurophysiological phenomena were not directly and unequivocally related to the perception of ongoing pain.

In this study, we investigated the neurophysiological encoding of ongoing, tonic pain by using electroencephalography (EEG). We specifically combined tonic painful heat stimuli and a continuous pain rating procedure with time–frequency analyses of EEG recordings to relate time courses of subjective pain intensity and objective stimulus intensity to those of frequency-specific brain activity. We further compared the encoding of tonic pain to that of brief painful stimuli.

## Materials and Methods

### Subjects

Forty-one healthy subjects (age 26 ± 6 years [mean ± standard deviation]; 22 females) participated in the experiment. All subjects gave written informed consent. The study was approved by the ethics committee of the Medical Faculty of the Technische Universität München and conducted in conformity with the Declaration of Helsinki.

### Paradigm

The paradigm comprised 3 conditions: The main *tonic pain* condition, a *visual control*, and a *phasic pain* condition.

**Figure 1.**
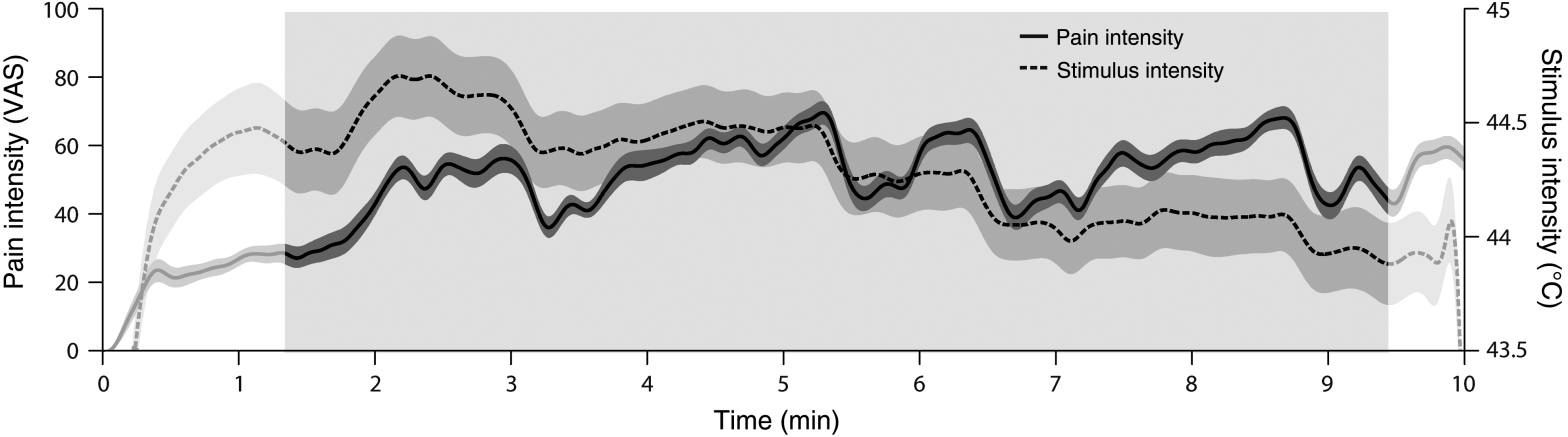
Time courses of pain intensity and stimulus intensity. Group mean time courses of subjective pain intensity and objective stimulus intensity during tonic painful heat stimulation of the left hand. Pain intensity was continuously rated on a VAS anchored at *no pain* and *worst tolerable pain.* Shaded areas around the curves depict the standard error of the mean. The light gray section indicates the time window used for the analysis. For display purposes, mean time courses were low-pass filtered at 0.1 Hz.

In the main *tonic pain* condition, tonic painful heat stimuli were delivered by a thermode (TSA-II, Medoc, Israel) to the dorsum of the subject's left hand for a duration of 10 min. Subjects were instructed to continuously rate the perceived pain intensity on a visual analog scale (VAS) ranging from 0 to 100 and anchored at *no pain* and *worst tolerable pain* using a custom-built finger-span device implemented as a potentiometer controlled by their right hand. The scale was simultaneously presented on a screen by a vertical red bar, the length of which represented the current pain intensity rating. Stimulus intensity was continuously adjusted aiming to match the individual pain rating with a predefined time course of pain intensity ranging from VAS 30 to 70. This time course included phases of steady and changing pain intensity with VAS levels of 30, 40, 50, 60, and 70. The initial increase and the final decrease of stimulus and pain intensity were not included in the analysis, resulting in an 8-min time window for the analysis marked by the light gray-shaded section in Figure 1.

A *visual control* condition was performed to control for the sensory, motor, and attentional components of the continuous pain rating procedure (Baliki et al. 2006, 2011; Hashmi et al. 2013). The temporally inverted time course of the individual pain intensity ratings from the tonic pain condition was visually presented as variations of the red bar over time. Subjects were instructed to continuously rate the length of the vertical red bar using the finger-span device controlled by the right hand. No painful stimulation was applied. Thus, in the control condition, subjects did not rate the perceived pain intensity but the length of a visual bar, ensuring that the visual input and the motor components of the rating procedure were similar to the *tonic pain* condition.

In a further condition, the encoding of *phasic pain* was investigated. To this end, 75 brief thermal laser stimuli were applied to the dorsum of the left hand using a Tm:YAG laser (Starmedtec GmbH, Starnberg, Germany) with a wavelength of 1960 nm, a pulse duration of 1 ms, and a spot diameter of 5 mm. A distance pin mounted to the hand piece of the laser device ensured a constant distance between skin surface and laser device. Stimulation site was slightly varied after each stimulus to avoid tissue damage, and subjects were instructed to keep their eyes open. Three seconds after each stimulus, subjects were prompted by an auditory cue to provide verbal pain ratings on a numerical rating scale (NRS) ranging from 0 to 100 anchored at *no pain* and *worst tolerable pain*. Stimulus intensity was varied aiming at eliciting NRS ratings at the levels 30, 40, 50, 60, and 70. To this end, individual stimulation intensities were determined beforehand on the basis of 15 laser stimuli with random intensities using a regression analysis relating objective stimulation intensities to subjective pain ratings. To mimic the adaptive stimulation procedure of the tonic pain condition and to control for potential habituation and sensitization, laser intensities were adapted after 25 and 50 trials applying the same approach to the last 25 stimuli. The resulting mean stimulus intensity and pain intensity ratings were 517 ± 71 mJ and NRS 38 ± 20 (mean ± standard deviation).

To match the temporal structure of the *visual control* with the *tonic pain* condition, the *visual control* condition was performed after the *tonic pain* condition. Otherwise, the order of conditions was randomized across subjects. Subjects had a few minutes break in between the conditions. During the recordings, subjects were exposed to white noise through headphones to cancel out ambient noise.

### EEG Recordings and Preprocessing

During all conditions, EEG data were recorded using an electrode cap (Easycap, Herrsching, Germany). The electrode montage included 64 electrodes consisting of all 10–20 system electrodes and the additional electrodes Fpz, FCz, CPz, POz, Oz, Iz, AF3/4, F5/6, FC1/2/3/4/5/6, FT7/8/9/10, C1/2/5/6, CP1/2/3/4/5/6, TP7/8/9/10, P5/6, and PO1/2/9/10, plus 2 electrodes below the outer canthus of each eye. During the recording, the EEG was referenced to the FCz electrode, grounded at AFz, sampled at 5 kHz (0.1 µV resolution), and high-pass filtered at 0.5 Hz. The impedance was kept below 20 kΩ. Continuous pain ratings and stimulation intensities, that is, the temperature of the thermode, were fed into the EEG system and simultaneously recorded as additional channels with the same sampling frequency.

The raw EEG data were preprocessed using the BrainVision Analyzer software (Brain Products, Munich, Germany) including downsampling to 512 Hz, correcting for eye movements and muscle artifacts using independent component analysis (Jung et al. 2000), and transforming to the average reference. Subsequently, time frames exceeding an amplitude of 80 µV were rejected. For further artifact rejection, time–frequency analysis was performed as described below. For each condition, data were *z*-transformed for each frequency band across all time points and electrodes. Time frames exceeding a *z*-value of 2 in the gamma band (30–100 Hz) were considered as contaminated with artifacts and also excluded from further analysis. For the phasic pain condition, data were segmented into trials of −1 to 1.5 s with respect to the laser stimulus.

Due to poor data quality, data from 1 and 2 subjects had to be discarded in the tonic pain condition and the visual control condition, respectively.

### Time–Frequency Analysis

Time–frequency analyses were performed using custom programming on the basis of standard mathematical and signal analysis functions in Matlab (Mathworks, Natick, MA, USA). To decompose frequencies from raw EEG, we applied a sliding-window Hanning-tapered, short-time Fast Fourier Transformation. The window had a length of 512 data points (1 s) and was shifted in steps of 20 data points.

For further analyses of the tonic pain and visual control conditions, average power was computed for each time point in the following frequency bands: theta (4–7 Hz), alpha (8–13 Hz), beta (14–29 Hz), and gamma (30–100 Hz). In the tonic pain condition, the initial increase and the final decrease of stimulus and pain intensity were discarded resulting in an 8-min analysis window (Fig. 1). The same time window was used for the analysis of the visual control condition.

For further analyses of the phasic pain condition, power was averaged across time–frequency windows of interest, which were based on previous studies (Mouraux et al. 2003; Ploner et al. 2006a, 2006b; Gross et al. 2007; Hauck et al. 2007; Zhang et al. 2012) and covered the strongest laser-induced responses: theta, 4–8 Hz, 0.15–0.35 s; alpha, 9–13 Hz, 0.47–0.70 s; beta, 14–29 Hz, 0.30–0.50 s; gamma, 76–86 Hz, 0.20–0.30 s (Fig. 4*A*).

### Relationship Between Pain/Stimulus Intensity and Brain Activity

The focus of the study was to investigate the neurophysiological encoding of subjective pain intensity and objective stimulus intensity during tonic painful stimulation. To this end, we fitted linear mixed models (LMMs) to the data from the tonic pain condition using custom scripts in Matlab. For each electrode and frequency band, pain/stimulus intensity was taken as a response variable and brain activity as a predictor. To account for the different subjects, we included a random intercept and random slope. As an overall decrease of stimulus intensity over time was observed, this sensitization effect was removed by detrending the time courses of stimulus intensity before the LMM analysis. These analyses yielded a statistical estimate of the strength of the relationship between brain activity and pain/stimulus intensity. Threshold of statistical significance was set at *P* < 0.05. False discovery rate (FDR) correction was performed across electrodes to control for type I error (Genovese et al. 2002). To more closely determine the frequency distribution of significant relations, we averaged time–frequency-transformed brain activity across those electrodes showing significant effects and repeated the LMM fitting for those electrodes only but now frequency-resolved in steps of 1 Hz between 1 and 100 Hz.

As control analyses, we correspondingly fitted LMM on the basis of brain activity and the bar length rating from the visual control condition as well as on the basis of brain activity and the temporally inverted time courses of pain intensity from the tonic pain condition. FDR correction for multiple testing was performed across electrodes. These analyses controlled for the sensory, motor, and attentional components of the continuous pain rating procedure and the autocorrelation of the data, respectively. In addition, we tested the specificity of the positive relation between pain intensity and gamma oscillations in the tonic pain condition. At those electrodes with a significant effect, we compared the relation between tonic pain and gamma oscillations with the relation between the visual rating and gamma oscillations and with the relation between the inverted time course of tonic pain intensity and gamma oscillations. Similar LMMs as before were fitted, now also including the main effect of condition (tonic pain vs. visual control/tonic pain vs. inverted pain) in addition to the main effect of brain activity. Again, to account for the different subjects, those effects were modeled as random effects.

Finally, we calculated LMM to assess the encoding of phasic pain as described previously (Schulz et al. 2011). Based on the phasic pain condition, this analysis related objective stimulus intensities and subjective pain intensity ratings to the time–frequency-transformed and baseline-corrected laser-induced brain activity on a single-trial basis. Again, FDR correction for multiple testing was performed across electrodes.

### Source Analysis

**Figure 2.**
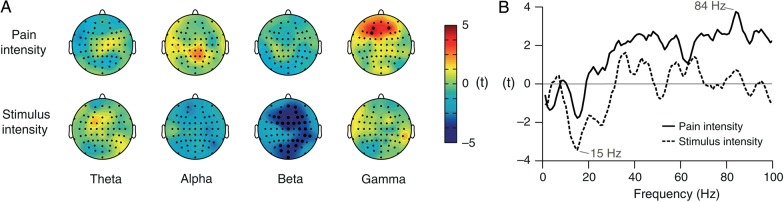
Neurophysiological encoding of pain intensity and stimulus intensity during tonic pain. (*A*) Topographies of the relationship between pain intensity/stimulus intensity and brain activity as assessed by LMMs. LMMs were calculated for theta (4–7 Hz), alpha (8–13 Hz), beta (14–29 Hz), and gamma (30–100 Hz) frequencies. Positive and negative relationships are depicted by warm and cold colors, respectively. Electrodes with a significant relationship between pain/stimulus intensity and brain activity after FDR correction for multiple testing are marked by bold black dots. (*B*) Frequency spectra of the relationship between pain/stimulus intensity and brain activity. LMMs were calculated for frequencies between 1 and 100 Hz for electrodes which had shown a significant relationship between pain/stimulus intensity and brain activity as displayed in (*A*). The strongest (positive) relationship between pain intensity and brain activity was observed at 84 Hz, and the strongest (negative) relationship between stimulus intensity and brain activity was found at 15 Hz.

On the electrode level, 2 significant relationships between brain activity and pain/stimulus intensity in the tonic pain condition were identified: A positive relationship between pain intensity and gamma oscillations and a negative relationship between stimulus intensity and beta oscillations (Fig. 2). To localize the sources of these relationships, we used the dynamic imaging of coherent sources (DICS) beamforming approach (Gross et al. 2001) implemented in the open-source Matlab toolbox FieldTrip (Oostenveld et al. 2011). The leadfield matrix was computed for a 10-mm 3D grid using the boundary element method volume conduction model, derived from the MNI template brain provided by FieldTrip. Cross-spectral density matrices were computed separately for beta and gamma frequencies using a multitaper time–frequency analysis on the EEG electrode data. By using DICS, a spatial filter was created based on cross-spectral density matrices of the entire time course. Electrode-level time–frequency data were then multiplied by this filter to obtain time courses of power for each grid point and the selected frequencies. Subsequently, LMMs were fitted as described before, now quantifying the relationship between brain activity at gamma/beta frequencies and pain/stimulus intensity on source level. For display purposes, data were downsampled to 2 × 2 × 2 mm^3^, smoothed with a 12-mm Gaussian kernel, and thresholded at *t* = 2.4 and −3.0.

## Results

### Neurophysiological Encoding of Tonic Pain

Figure 1 shows the group mean time courses of objective stimulus intensity and subjective pain intensity in the main *tonic pain* condition. The time courses show an initial increase of both measures followed by a phase of slow changes and a final decrease. Furthermore, an overall decrease of stimulus intensity over time was observed indicating a sensitization to the stimulation. The initial increase and the final decrease of stimulus and pain intensity were not included in the analysis, resulting in an 8-min time window for the analysis marked by the light gray-shaded section in Figure 1. During this time window, group mean stimulus intensity and pain intensity were 44 ± 0.8 °C and VAS 47 ± 25 (mean ± standard deviation), respectively.

We first determined brain activity that encodes the subjective perception of tonic pain. To this end, EEG data were time–frequency-transformed and time courses of brain activity were computed for different frequency bands. These frequency-specific time courses of brain activity were related to time courses of subjective pain intensity by calculating LMMs. This analysis yielded a statistical estimate of the strength of the relationship between brain activity and subjective pain intensity for each electrode and frequency band. The upper row of Figure 2*A* shows the topographies of this relationship for theta (4–7 Hz), alpha (8–13 Hz), beta (14–29 Hz), and gamma (30–100 Hz) frequencies. The results show that neuronal gamma oscillations at frontal electrodes encoded the subjective intensity of tonic pain (*t*_max_ = 4.1 at electrode F3). Single-subject data indicate that this relationship was not driven by outliers (Supplementary Table 1). The peak frequency of this positive relationship between pain intensity and gamma oscillations was 84 Hz (Fig. 2*B*). No significant relationship between subjective pain intensity and brain activity was observed at theta, alpha, or beta frequencies.

We next investigated the cerebral encoding of objective stimulus intensity during tonic painful stimulation. We now related time courses of stimulus intensity, that is, stimulation temperature to frequency-specific brain activity using LMM. In contrast to the encoding of pain intensity by frontal gamma oscillations, we found that stimulus intensity was negatively related to beta oscillations (Fig. 2*A*, lower row). This relationship was observed at an extended array of EEG electrodes lateralized to the right side, that is, contralateral to stimulus application (*t*_max_ = 4.7 at electrode Fz). The peak frequency of this negative relationship between stimulus intensity and beta oscillations was 15 Hz (Fig. 2*B*). The relation was observed after removing effects due to sensitization by detrending the data before analysis. No significant relationship between stimulus intensity and brain activity was observed at theta, alpha, or gamma frequencies. We thus observed dissociation between the cerebral encoding of subjective pain intensity and objective stimulus intensity during tonic painful stimulation.

### Control Conditions

**Figure 3.**
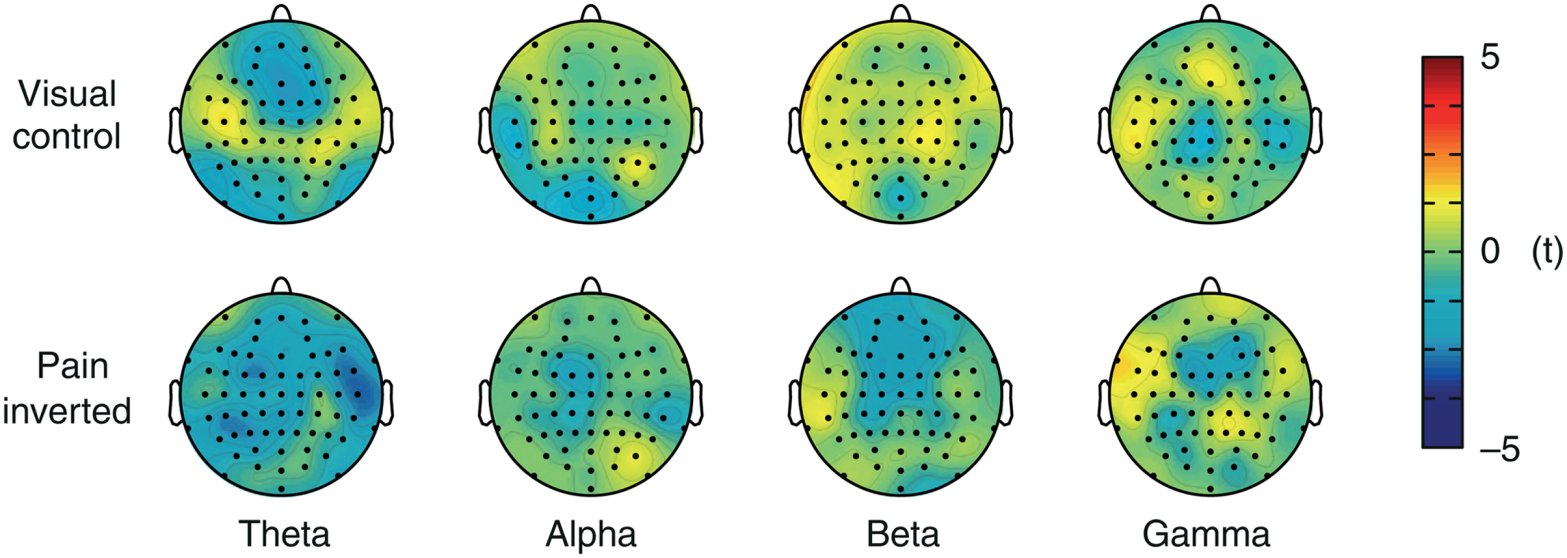
Control conditions. Topographies of the relationship between brain activity and bar length rating in the visual control condition (upper row) and between brain activity and the inverted time course of pain intensity in the tonic pain condition (lower row) as assessed by LMMs. LMMs were calculated for theta (4–7 Hz), alpha (8–13 Hz), beta (14–29 Hz), and gamma (30–100 Hz) frequencies. Positive and negative relationships are depicted by warm and cold colors, respectively. No significant relationships were observed after FDR correction for multiple testing.

To control for the visual and motor components of the continuous pain rating procedure, we performed a visual control condition (Baliki et al. 2006, 2011; Hashmi et al. 2013), which did not include the rating of pain but of the length of a visual bar, whereas the visual input and the motor components of the rating procedure were similar to the main condition. The results of an LMM analysis relating the rating of the bar length to frequency-specific brain activity did not show a significant relationship at any frequency band (Fig. 3, upper row). Furthermore, an additional LMM analysis was performed, which specifically assessed differences between the tonic pain and visual control condition. This analysis focused on those frontal electrodes where a significant relationship between gamma oscillations and pain intensity in the tonic pain condition had been observed. The results confirm that the relationship between gamma oscillations and pain intensity differs significantly from that between gamma oscillations and the visual rating (*t*_max_ = 2.7 at electrode F3). This analysis indicates that the encoding of pain intensity by frontal gamma oscillations does not reflect an encoding of visual information or motor components of the rating procedure.

Finally, we performed a control analysis for the autocorrelation of the data. To this end, we calculated another LMM analysis of the tonic pain condition, now using the temporally inverted time courses of pain intensity ratings. The results did not show a significant relationship between the inverted time courses of pain intensity and brain activity at any frequency or electrode (Fig. 3, lower row). In addition, at frontal electrodes, where a significant relationship between gamma oscillations and pain intensity had been observed in the main analysis, an extended LMM analysis confirmed a significant difference between the encoding of pain intensity and inverted pain intensity (*t*_max_ = 2.8 at electrode F3).

### Neurophysiological Encoding of Phasic Pain

**Figure 4.**
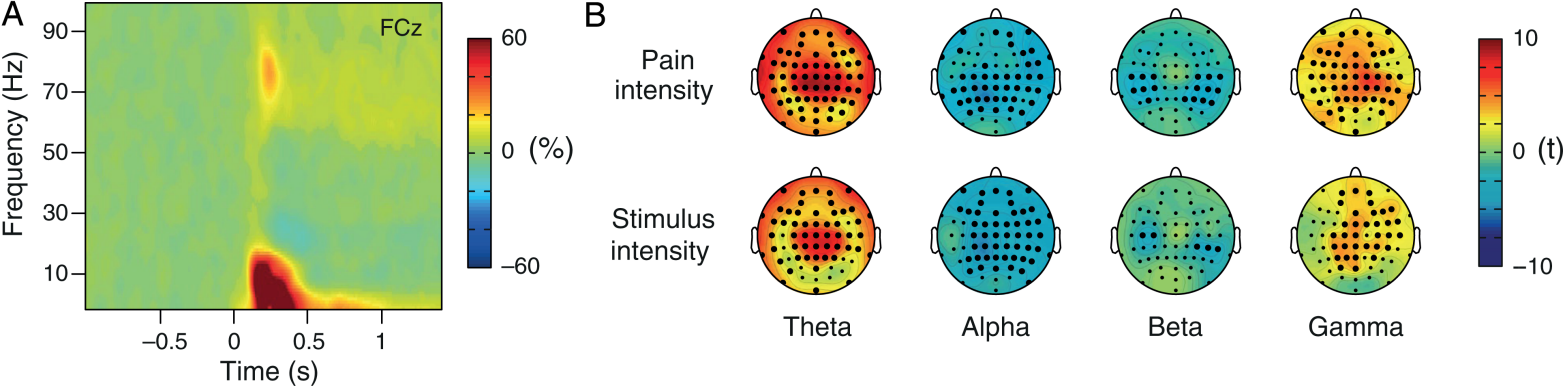
Neurophysiological encoding of pain intensity and stimulus intensity during phasic pain. (*A*) Group mean time–frequency representation of neuronal responses to phasic painful stimuli at electrode FCz. Neuronal responses are displayed as percent signal change relative to a pre-stimulus baseline (−1000 to 0 ms). Positive and negative signal changes are depicted by warm and cold colors, respectively. (*B*) Topographies of the relationship between stimulus/pain intensity and brain activity as assessed by LMMs. LMMs were calculated for time–frequency windows defined from previous studies (theta, 4–8 Hz, 0.15–0.35 s; alpha, 9–13 Hz, 0.47–0.70 s; beta, 14–29 Hz, 0.30–0.50 s; and gamma, 76–86 Hz, 0.20–0.30 s). Positive and negative relationships are depicted by warm and cold colors, respectively. Electrodes with a significant relationship between pain/stimulus intensity and brain activity after FDR correction for multiple testing are marked by bold black dots.

We next compared the neurophysiological encoding of tonic pain with the encoding of phasic pain. To this end, we applied 75 brief thermal laser stimuli to the subjects' left hand, obtained single-trial pain ratings, recorded EEG, and determined stimulus-locked time–frequency-transformed brain activity (Fig. 4*A*). We related brain activity to objective stimulus intensity and subjective pain intensity by calculating LMM on a single-trial basis. Figure 4*B* shows topographies of these relationships at theta, alpha, beta, and gamma frequencies. For both stimulus intensity and pain intensity, we found positive relationships to brain activity at theta (*t*_max_ = 8.2 and 9.0 at electrodes CP2 and CPz, respectively) and gamma frequencies (*t*_max_ = 4.7 and 6.6 at electrodes FCz and C2, respectively), and negative relationships to brain activity at alpha (*t*_max_ = −5.0 and −5.1 at electrodes CP1 and Pz, respectively) and beta (*t*_max_ = −3.2 and −4.2 at electrodes CP6 and FT8, respectively) frequencies. The topographies show qualitatively similar widespread patterns for the encoding of both stimulus intensity and pain intensity. They further indicate that gamma oscillations encoding the subjective intensity of phasic pain are lateralized to the right side, that is, contralateral to stimulus application.

As the temporal structure and signal-to-noise ratio of phasic and tonic pain fundamentally differ, we compared the encoding of both phenomena qualitatively on a descriptive level. The comparison shows 3 fundamental differences. First, for tonic pain, we observed dissociation between the encoding of stimulus intensity and pain intensity. In contrast, for phasic pain, we found qualitatively similar patterns for the encoding of both stimulus intensity and pain intensity. Secondly, the subjective intensity of phasic pain was encoded by brain activity at different frequencies, whereas that of tonic pain was encoded by prefrontal gamma oscillations only. Thirdly, the subjective perception of phasic pain was encoded by right-lateralized activity at central electrodes, whereas the subjective perception of tonic pain was encoded by gamma activity recorded from mid-frontal electrodes. The direct comparison between tonic and phasic pain, thus, reveals that the cerebral representation of both phenomena differs fundamentally.

### Brain Sources Encoding Tonic Pain

**Figure 5.**
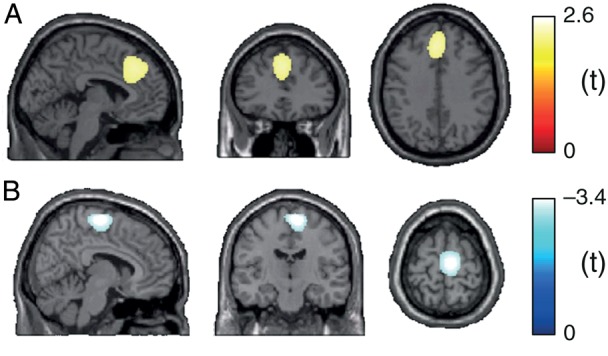
Brain sources encoding tonic pain. Locations of (*A*) the strongest relationship between subjective pain intensity and brain activity in the gamma band (30–100 Hz) and (*B*) the strongest relationship between objective stimulus intensity and brain activity in the beta band (14–29 Hz) as assessed by LMM in source space. Positive and negative relationships are depicted by warm and cold colors, respectively. MNI coordinates of strongest relationships (peak locations) were −4, 34, 36 in (*A*) and 8, −16, 68 in (*B*).

We finally determined the location of sources encoding tonic pain in the brain. Based on the results of the electrode-based analyses, we focused this analysis on the relationship between subjective pain intensity and brain activity at gamma frequencies, and the relationship between objective stimulus intensity and brain activity at beta frequencies. The results revealed that the area with the strongest relationship between pain intensity and gamma oscillations was located in the mid-prefrontal cortex (Fig. 5) adjacent to the premotor and cingulate cortices. The strongest relationship between stimulus intensity and beta activity was found in the superior frontal cortex. The location and lateralization of this latter relationship to the right hemisphere suggests a significant contribution of sensorimotor areas.

## Discussion

In the present study, we investigated the neurophysiological encoding of ongoing, tonic pain in humans. Our results show that, during longer-lasting painful stimulation, the encoding of subjective pain intensity dissociates from that of objective stimulus intensity. Subjective pain intensity was specifically encoded by gamma oscillations recorded over medial prefrontal cortex, whereas objective stimulus intensity was negatively related to beta oscillations lateralized to the hemisphere contralateral to stimulation. Furthermore, a direct comparison reveals that already at a timescale of minutes the encoding of tonic pain differs fundamentally from that of brief painful stimuli.

Previous evidence about the neurophysiological encoding of ongoing pain is sparse. Our observation that objective stimulus intensity was inversely related to neuronal oscillations at low beta frequencies close to the alpha frequency band is in good agreement with previous studies, which mostly showed a suppression of beta and alpha oscillations during ongoing pain (Chen and Rappelsberger 1994; Ferracuti et al. 1994; Chang et al. 2002; Dowman et al. 2008; Nir et al. 2012; Shao et al. 2012; Peng et al. 2014). However, our study provides the first demonstration that objective stimulus intensity, but not subjective pain intensity, of tonic pain is encoded in suppression of alpha/beta oscillations. This suppression might reflect the alerting function of pain (Ploner et al. 2006a, 2006b), which increases the excitability of somatosensory cortex (Ploner et al. 2006a, 2006b) and supports the attentional integration of pain (Palva and Palva 2011; May et al. 2012; Hu et al. 2013). Even fewer studies have investigated neuronal gamma oscillations during tonic pain (Veerasarn and Stohler 1992; Dowman et al. 2008; Peng et al. 2014). All of these results showed increases of gamma oscillations, but only a single study related them to pain perception (Peng et al. 2014). However, this study did not consistently observe gamma oscillations during tonic pain and did not disentangle subjective pain intensity and objective stimulus intensity. Therefore, it could not establish an unequivocal link between neuronal gamma oscillations and ongoing pain.

Comparatively few functional imaging studies have investigated the cerebral representation of ongoing pain. They observed signal changes in brain areas that are also implicated in the processing of brief experimental pain, that is, in the thalamus and somatosensory, insular, and cingulate cortices (Di Piero et al. 1994; Hsieh et al. 1995; Derbyshire and Jones 1998; Schreckenberger et al. 2005; Owen et al. 2010; Wasan et al. 2011). A recent seminal series of investigations pursued a novel approach to the cerebral encoding of ongoing pain (Baliki et al. 2006, 2011; Hashmi et al. 2013). The authors conceptualized ongoing pain as a dynamic process, obtained continuous pain ratings, and performed advanced analyses of fMRI data to relate the dynamics of ongoing pain to brain activity. The results revealed that ongoing pain at a timescale of months and years is closely related to BOLD activity in the medial prefrontal cortex. Based on these observations, the authors proposed a shift from brain circuits associated with sensory processes to emotional circuits during the development of chronic pain (Hashmi et al. 2013). Here, we adapted this approach to neurophysiological data and found that, already on a timescale of minutes, the subjective perception of ongoing, tonic pain is encoded in the medial prefrontal cortex. We further found a frequency-specific neurophysiological signature of tonic pain, that is, neuronal gamma oscillations. Specifically, the amplitude of gamma oscillations in the medial prefrontal cortex encoded the subjective perception of pain but not objective stimulus intensity.

Recently, brief painful stimuli have been shown to induce gamma oscillations in somatosensory cortices (Gross et al. 2007; Hauck et al. 2007; Tiemann et al. 2010; Schulz et al. 2011; Zhang et al. 2012; Rossiter et al. 2013), which likely reflect the local processing of sensory information (Donner and Siegel 2011) in the somatosensory cortex. In the physiological condition of brief or acute pain, these sensory processes might faithfully translate into the subjective perception of pain (Garcia-Larrea and Peyron 2013). However, we here observed that, during longer-lasting painful stimulation, the encoding of the subjective perception of pain dissociates from that of objective sensory information. Specifically, the perception of tonic pain was not encoded by gamma oscillations over the somatosensory cortex but over the medial prefrontal cortex close to premotor and cingulate cortices. This is in agreement with studies in monkeys which showed that neural activity in the medial prefrontal cortex is more closely related to the subjective perception of somatosensory stimuli than to objective stimulus intensity (Romo and de Lafuente 2013). Furthermore, premotor and cingulate cortices are well known to be critically involved in the cerebral processing of pain. The cingulate cortex is an integrative brain area at a high level of the pain processing hierarchy (Garcia-Larrea and Peyron 2013). It has been proposed to integrate sensory, emotional, and cognitive information (Shackman et al. 2011) in order to assign behavioral relevance, or salience, to events and states (Legrain et al. 2011; Borsook et al. 2013). This integrated salience signal might represent the basis for the ultimate biological function of pain, that is, the choice and guidance of an appropriate behavioral response (Shackman et al. 2011). The encoding of tonic pain by prefrontal gamma oscillations might therefore indicate that the subjective perception of ongoing pain is more dependent on contextual, integrative, and evaluative-emotional than on sensory processes, which more strongly determine the perception of brief painful stimuli. Furthermore, magnetic resonance spectroscopy has shown that tonic pain yields increased GABA concentrations in the cingulate cortex (Kupers et al. 2009). As gamma oscillations depend on GABAergic neurotransmission (Buzsaki and Wang 2012), our results are compatible with the hypothesis that GABAergic dysfunction induces abnormal gamma oscillations, which might result in ongoing pain during chronic pain states (Barr et al. 2013).

Several potentially confounding factors need to be considered. First, neuronal gamma activity can be confounded by muscle activity. However, muscle activity is typically strongest at the most frontal and lateral electrodes (Goncharova et al. 2003), whereas we found the strongest relationship between perception and gamma oscillations at midline electrodes. We, moreover, applied an independent component analysis-based correction for muscle artifacts (Jung et al. 2000), and a source analysis based on beamforming (which is comparatively robust against muscle artifacts, Hipp and Siegel 2013) confirmed our results. Secondly, our control conditions control for visual, motor, and attentional effects but not for salience. The observed encoding of pain intensity is therefore not necessarily pain-specific, but may reflect the salience of pain. Thirdly, we applied a paradigm with slow changes in stimulus intensity. This approach conceptualizes tonic pain as a dynamic process, which corresponds to recent notions on the dynamics of chronic pain (Baliki et al. 2006, 2011; Foss et al. 2006; Hashmi et al. 2013) and allows for directly relating pain perception to brain activity. The approach, however, implies that our observations do not necessarily generalize to conditions of ongoing pain with different dynamics, particularly to the years and decades of ongoing pain in chronic pain states. Our study nevertheless shows that the encoding of pain over a timescale of minutes already differs substantially from that of brief painful stimuli.

Taken together, we observed that tonic pain is encoded by gamma oscillations in the medial prefrontal cortex. This encoding pattern differs fundamentally from that of objective stimulus intensity and from the encoding of brief experimental pain. These results extend current concepts of the brain mechanisms of pain to the clinically relevant state of ongoing pain. They specifically suggest that, already on a timescale of minutes, the perception of tonic pain depends on emotional-evaluative circuits rather than on sensory circuits. Moreover, the encoding of tonic pain by prefrontal gamma oscillations in EEG recordings might help to identify a spatially and frequency-specific functional brain marker of ongoing pain, which could be useful for the diagnosis and therapy of chronic pain (Davis et al. 2012). Specifically, it could serve as an interesting target for EEG-based neurofeedback approaches (Jensen et al. 2014).

## Supplementary Material

Supplementary material can be found at: http://www.cercor.oxfordjournals.org/.

## Funding

The study was supported by the Deutsche Forschungsgemeinschaft (PL 321/10-1, PL 321/11-1, and RTG 1373) and the Else Kröner-Fresenius-Stiftung (2011_A82). J.G. is supported by the Wellcome Trust (098433). Funding to pay the Open Access publication charges for this article was provided by Wellcome Trust.

## Supplementary Material

Supplementary Data

## References

[BHV043C1] Apkarian AV Bushnell MC Schweinhardt P 2013 Representation of pain in the brain. In: McMahon SB KoltzenburgTracey MI Turk DC, editors. Wall and Melzack's Textbook of pain. 6th ed Philadelphia: Elsevier p. 111–128.

[BHV043C2] Baliki MN Baria AT Apkarian AV 2011 The cortical rhythms of chronic back pain. J Neurosci. 31:13981–13990. 21957259 10.1523/JNEUROSCI.1984-11.2011 PMC3214084

[BHV043C3] Baliki MN Chialvo DR Geha PY Levy RM Harden RN Parrish TB Apkarian AV 2006 Chronic pain and the emotional brain: specific brain activity associated with spontaneous fluctuations of intensity of chronic back pain. J Neurosci. 26:12165–12173. 17122041 10.1523/JNEUROSCI.3576-06.2006 PMC4177069

[BHV043C4] Barr MS Farzan F Davis KD Fitzgerald PB Daskalakis ZJ 2013 Measuring GABAergic inhibitory activity with TMS-EEG and its potential clinical application for chronic pain. J Neuroimmune Pharmacol. 8:535–546. 22744222 10.1007/s11481-012-9383-y

[BHV043C5] Borsook D Edwards R Elman I Becerra L Levine J 2013 Pain and analgesia: the value of salience circuits. Prog Neurobiol. 104:93–105. 23499729 10.1016/j.pneurobio.2013.02.003 PMC3644802

[BHV043C6] Breivik H Collett B Ventafridda V Cohen R Gallacher D 2006 Survey of chronic pain in Europe: prevalence, impact on daily life, and treatment. Eur J Pain. 10:287–333. 16095934 10.1016/j.ejpain.2005.06.009

[BHV043C7] Buzsaki G Wang XJ 2012 Mechanisms of gamma oscillations. Annu Rev Neurosci. 35:203–225. 22443509 10.1146/annurev-neuro-062111-150444 PMC4049541

[BHV043C8] Chang PF Arendt-Nielsen L Chen AC 2002 Dynamic changes and spatial correlation of EEG activities during cold pressor test in man. Brain Res Bull. 57:667–675. 11927371 10.1016/s0361-9230(01)00763-8

[BHV043C9] Chen AC Rappelsberger P 1994 Brain and human pain: topographic EEG amplitude and coherence mapping. Brain Topogr. 7:129–140. 7696090 10.1007/BF01186771

[BHV043C10] Davis KD Racine E Collett B 2012 Neuroethical issues related to the use of brain imaging: can we and should we use brain imaging as a biomarker to diagnose chronic pain? Pain. 153:1555–1559. 22464695 10.1016/j.pain.2012.02.037

[BHV043C11] Derbyshire SW Jones AK 1998 Cerebral responses to a continual tonic pain stimulus measured using positron emission tomography. Pain. 76:127–135. 9696465 10.1016/s0304-3959(98)00034-7

[BHV043C12] Di Piero V Ferracuti S Sabatini U Pantano P Cruccu G Lenzi GL 1994 A cerebral blood flow study on tonic pain activation in man. Pain. 56:167–173. 8008407 10.1016/0304-3959(94)90091-4

[BHV043C13] Donner TH Siegel M 2011 A framework for local cortical oscillation patterns. Trends Cogn Sci. 15:191–199. 21481630 10.1016/j.tics.2011.03.007

[BHV043C14] Dowman R Rissacher D Schuckers S 2008 EEG indices of tonic pain-related activity in the somatosensory cortices. Clin Neurophysiol. 119:1201–1212. 18337168 10.1016/j.clinph.2008.01.019 PMC2676940

[BHV043C15] Ferracuti S Seri S Mattia D Cruccu G 1994 Quantitative EEG modifications during the Cold Water Pressor Test: hemispheric and hand differences. Int J Psychophysiol. 17:261–268. 7806469 10.1016/0167-8760(94)90068-x

[BHV043C16] Foss JM Apkarian AV Chialvo DR 2006 Dynamics of pain: fractal dimension of temporal variability of spontaneous pain differentiates between pain states. J Neurophysiol. 95:730–736. 16282201 10.1152/jn.00768.2005

[BHV043C17] Garcia-Larrea L Frot M Valeriani M 2003 Brain generators of laser-evoked potentials: from dipoles to functional significance. Neurophysiol Clin. 33:279–292. 14678842 10.1016/j.neucli.2003.10.008

[BHV043C18] Garcia-Larrea L Peyron R 2013 Pain matrices and neuropathic pain matrices: a review. Pain. 154 (Suppl 1):S29–S43. 24021862 10.1016/j.pain.2013.09.001

[BHV043C19] Genovese CR Lazar NA Nichols T 2002 Thresholding of statistical maps in functional neuroimaging using the false discovery rate. Neuroimage. 15:870–878. 11906227 10.1006/nimg.2001.1037

[BHV043C20] Goncharova II McFarland DJ Vaughan TM Wolpaw JR 2003 EMG contamination of EEG: spectral and topographical characteristics. Clin Neurophysiol. 114:1580–1593. 12948787 10.1016/s1388-2457(03)00093-2

[BHV043C21] Gross J Kujala J Hamalainen M Timmermann L Schnitzler A Salmelin R 2001 Dynamic imaging of coherent sources: studying neural interactions in the human brain. Proc Natl Acad Sci USA. 98:694–699. 11209067 10.1073/pnas.98.2.694 PMC14650

[BHV043C22] Gross J Schnitzler A Timmermann L Ploner M 2007 Gamma oscillations in human primary somatosensory cortex reflect pain perception. PLoS Biol. 5:e133. 17456008 10.1371/journal.pbio.0050133 PMC1854914

[BHV043C23] Hashmi JA Baliki MN Huang L Baria AT Torbey S Hermann KM Schnitzer TJ Apkarian AV 2013 Shape shifting pain: chronification of back pain shifts brain representation from nociceptive to emotional circuits. Brain. 136:2751–2768. 23983029 10.1093/brain/awt211 PMC3754458

[BHV043C24] Hauck M Lorenz J Engel AK 2007 Attention to painful stimulation enhances gamma-band activity and synchronization in human sensorimotor cortex. J Neurosci. 27:9270–9277. 17728441 10.1523/JNEUROSCI.2283-07.2007 PMC6673131

[BHV043C25] Hipp JF Siegel M 2013 Dissociating neuronal gamma-band activity from cranial and ocular muscle activity in EEG. Front Hum Neurosci. 7:338. 23847508 10.3389/fnhum.2013.00338 PMC3706727

[BHV043C26] Hsieh JC Belfrage M Stone-Elander S Hansson P Ingvar M 1995 Central representation of chronic ongoing neuropathic pain studied by positron emission tomography. Pain. 63:225–236. 8628589 10.1016/0304-3959(95)00048-W

[BHV043C27] Hu L Peng W Valentini E Zhang Z Hu Y 2013 Functional features of nociceptive-induced suppression of alpha band electroencephalographic oscillations. J Pain. 14:89–99. 23273836 10.1016/j.jpain.2012.10.008

[BHV043C28] Jensen MP Day MA Miro J 2014 Neuromodulatory treatments for chronic pain: efficacy and mechanisms. Nat Rev Neurol. 10:167–178. 24535464 10.1038/nrneurol.2014.12 PMC5652321

[BHV043C29] Jung TP Makeig S Humphries C Lee TW McKeown MJ Iragui V Sejnowski TJ 2000 Removing electroencephalographic artifacts by blind source separation. Psychophysiology. 37:163–178. 10731767

[BHV043C30] Kupers R Danielsen ER Kehlet H Christensen R Thomsen C 2009 Painful tonic heat stimulation induces GABA accumulation in the prefrontal cortex in man. Pain. 142:89–93. 19167811 10.1016/j.pain.2008.12.008

[BHV043C31] Legrain V Iannetti GD Plaghki L Mouraux A 2011 The pain matrix reloaded: a salience detection system for the body. Prog Neurobiol. 93:111–124. 21040755 10.1016/j.pneurobio.2010.10.005

[BHV043C32] May ES Butz M Kahlbrock N Hoogenboom N Brenner M Schnitzler A 2012 Pre- and post-stimulus alpha activity shows differential modulation with spatial attention during the processing of pain. Neuroimage. 62:1965–1974. 22659486 10.1016/j.neuroimage.2012.05.071

[BHV043C33] Mouraux A Guerit JM Plaghki L 2003 Non-phase locked electroencephalogram (EEG) responses to CO_2_ laser skin stimulations may reflect central interactions between A-delta- and C-fibre afferent volleys. Clin Neurophysiol. 114:710–722. 12686279 10.1016/s1388-2457(03)00027-0

[BHV043C34] Nir RR Sinai A Moont R Harari E Yarnitsky D 2012 Tonic pain and continuous EEG: prediction of subjective pain perception by alpha-1 power during stimulation and at rest. Clin Neurophysiol. 123:605–612. 21889398 10.1016/j.clinph.2011.08.006

[BHV043C35] Oostenveld R Fries P Maris E Schoffelen JM 2011 FieldTrip: open source software for advanced analysis of MEG, EEG, and invasive electrophysiological data. Comput Intell Neurosci. 2011:156869. 21253357 10.1155/2011/156869 PMC3021840

[BHV043C36] Owen DG Clarke CF Ganapathy S Prato FS St Lawrence KS 2010 Using perfusion MRI to measure the dynamic changes in neural activation associated with tonic muscular pain. Pain. 148:375–386. 19914778 10.1016/j.pain.2009.10.003

[BHV043C37] Palva S Palva JM 2011 Functional roles of alpha-band phase synchronization in local and large-scale cortical networks. Front Psychol. 2:204. 21922012 10.3389/fpsyg.2011.00204 PMC3166799

[BHV043C38] Peng W Hu L Zhang Z Hu Y 2014 Changes of spontaneous oscillatory activity to tonic heat pain. PLoS ONE. 9:e91052. 24603703 10.1371/journal.pone.0091052 PMC3946288

[BHV043C39] Ploner M Gross J Timmermann L Pollok B Schnitzler A 2006a Oscillatory activity reflects the excitability of the human somatosensory system. Neuroimage. 32:1231–1236. 16854599 10.1016/j.neuroimage.2006.06.004

[BHV043C40] Ploner M Gross J Timmermann L Pollok B Schnitzler A 2006b Pain suppresses spontaneous brain rhythms. Cereb Cortex. 16:537–540. 16033927 10.1093/cercor/bhj001

[BHV043C41] Romo R de Lafuente V 2013 Conversion of sensory signals into perceptual decisions. Prog Neurobiol. 103:41–75. 22472964 10.1016/j.pneurobio.2012.03.007

[BHV043C42] Rossiter HE Worthen SF Witton C Hall SD Furlong PL 2013 Gamma oscillatory amplitude encodes stimulus intensity in primary somatosensory cortex. Front Hum Neurosci. 7:362. 23874282 10.3389/fnhum.2013.00362 PMC3711008

[BHV043C43] Schreckenberger M Siessmeier T Viertmann A Landvogt C Buchholz HG Rolke R Treede RD Bartenstein P Birklein F 2005 The unpleasantness of tonic pain is encoded by the insular cortex. Neurology. 64:1175–1183. 15824343 10.1212/01.WNL.0000156353.17305.52

[BHV043C44] Schulz E Tiemann L Schuster T Gross J Ploner M 2011 Neurophysiological coding of traits and states in the perception of pain. Cereb Cortex. 21:2408–2414. 21378113 10.1093/cercor/bhr027

[BHV043C45] Shackman AJ Salomons TV Slagter HA Fox AS Winter JJ Davidson RJ 2011 The integration of negative affect, pain and cognitive control in the cingulate cortex. Nat Rev Neurosci. 12:154–167. 21331082 10.1038/nrn2994 PMC3044650

[BHV043C46] Shao S Shen K Yu K Wilder-Smith EP Li X 2012 Frequency-domain EEG source analysis for acute tonic cold pain perception. Clin Neurophysiol. 123:2042–2049. 22538122 10.1016/j.clinph.2012.02.084

[BHV043C47] Tiemann L Schulz E Gross J Ploner M 2010 Gamma oscillations as a neuronal correlate of the attentional effects of pain. Pain. 150:302–308. 20558000 10.1016/j.pain.2010.05.014

[BHV043C48] Tracey I Mantyh PW 2007 The cerebral signature for pain perception and its modulation. Neuron. 55:377–391. 17678852 10.1016/j.neuron.2007.07.012

[BHV043C49] Veerasarn P Stohler CS 1992 The effect of experimental muscle pain on the background electrical brain activity. Pain. 49:349–360. 1408301 10.1016/0304-3959(92)90242-4

[BHV043C50] Wasan AD Loggia ML Chen LQ Napadow V Kong J Gollub RL 2011 Neural correlates of chronic low back pain measured by arterial spin labeling. Anesthesiology. 115:364–374. 21720241 10.1097/ALN.0b013e318220e880 PMC3286828

[BHV043C51] Zhang ZG Hu L Hung YS Mouraux A Iannetti GD 2012 Gamma-band oscillations in the primary somatosensory cortex—a direct and obligatory correlate of subjective pain intensity. J Neurosci. 32:7429–7438. 22649223 10.1523/JNEUROSCI.5877-11.2012 PMC6703598

