# Supplementary material for: Prefrontal Gamma Oscillations Encode Tonic Pain in Humans
Source: Cereb Cortex. 2015 Mar 8;25(11):4407–14. doi: 10.1093/cercor/bhv043 (PMC4816790; doi:10.1093/cercor/bhv043)
Supplement: Supplementary Data [file supp_25_11_4407__index.html]

Prefrontal Gamma Oscillations Encode Tonic Pain in Humans — Supplementary Data 

# Prefrontal Gamma Oscillations Encode Tonic Pain in Humans

## Supplementary Data

Supplementary Data

**Files in this Data Supplement:**

- Supplementary Data - Docx file
