# Supplementary material for: Prefrontal Gamma Oscillations Encode Tonic Pain in Humans
Source: Cereb Cortex. 2015 Mar 8;25(11):4407–14. doi: 10.1093/cercor/bhv043 (PMC4816790; doi:10.1093/cercor/bhv043)
Supplement: Supplementary Data [file supp_bhv043_bhv043supp.docx]

**Supplementary Table 1.** Single subject t-values quantifying the relationship between prefrontal gamma oscillations (averaged across electrodes AF3, AF4, and F3 as marked in Figure 2A), subjective pain intensity, and objective stimulus intensity.

| subject no. | gamma oscillations – pain intensity | gamma oscillations – stimulus intensity |
| --- | --- | --- |
| 1 | -1.6 | -11.7 |
| 2 | -0.4 | -4.0 |
| 3 | 20.0 | 1.1 |
| 4 | 4.6 | 0.2 |
| 5 | -1.1 | 6.5 |
| 6 | 0.1 | -0.7 |
| 7 | 0.1 | -1.9 |
| 8 | 6.0 | 7.3 |
| 9 | -2.2 | -2.3 |
| 10 | -3.8 | -2.0 |
| 11 | 22.1 | 11.4 |
| 12 | 2.9 | -11.3 |
| 13 | 6.0 | -6.1 |
| 14 | 2.8 | 2.3 |
| 15 | -1.1 | 1.0 |
| 16 | 13.8 | 0.3 |
| 17 | -0.5 | 13.0 |
| 18 | 6.1 | 3.7 |
| 19 | 19.7 | 8.9 |
| 20 | 33.7 | 9.9 |
| 21 | 10.9 | 17.3 |
| 22 | -6.2 | -3.6 |
| 23 | 24.7 | 4.5 |
| 24 | 35.4 | -2.7 |
| 25 | 9.6 | 6.2 |
| 26 | 2.1 | 1.2 |
| 27 | -4.2 | -1.8 |
| 28 | 2.3 | -12.7 |
| 29 | 5.5 | -10.7 |
| 30 | 0.3 | -0.8 |
| 31 | 4.2 | 0.6 |
| 32 | 25.8 | 6.1 |
| 33 | -7.0 | 1.1 |
| 34 | -3.7 | -0.7 |
| 35 | 0.6 | -0.7 |
| 36 | 6.9 | 8.7 |
| 37 | 31.6 | 7.3 |
| 38 | 6.5 | 0.3 |
| 39 | 3.0 | -0.4 |
| 40 | 16.5 | 7.4 |

Single subject t-values quantifying the relationship between prefrontal gamma oscillations and pain intensity were significantly different from zero (paired t-test, t = 4.1, p < 0.001). In contrast, t-values for the relationship between prefrontal gamma oscillations and stimulus intensity were not (paired t-test, t = 1.2, p = 0.23). In line with these results, the relationship between prefrontal gamma oscillations and pain intensity was significantly stronger than that between prefrontal gamma oscillations and stimulus intensity (paired t-test, t = 3.6, p < 0.001).
